# Inhibition of African swine fever virus protease by myricetin and myricitrin

**DOI:** 10.1080/14756366.2020.1754813

**Published:** 2020-04-17

**Authors:** Seri Jo, Suwon Kim, Dong Hae Shin, Mi-Sun Kim

**Affiliations:** College of Pharmacy and Graduates School of Pharmaceutical Sciences, Ewha W. University, Seoul, Republic of Korea

**Keywords:** African swine fever virus protease, antiviral, flavonoid, fret, inhibitory compounds

## Abstract

African swine fever (ASF) caused by the ASF virus (ASFV) is the most hazardous swine disease. Since a huge number of pigs have been slaughtered to avoid a pandemic spread, intense studies on the disease should be followed quickly. Recent studies reported that flavonoids have various antiviral activity including ASFV. In this report, ASFV protease was selected as an antiviral target protein to cope with ASF. With a FRET (Fluorescence resonance energy transfer) method, ASFV protease was assayed with a flavonoid library which was composed of sixty-five derivatives classified based on ten different scaffolds. Of these, the flavonols scaffold contains a potential anti-ASFV protease activity. The most prominent flavonol was myricetin with IC_50_ of 8.4 *μ*M. Its derivative, myricitrin, with the rhamnoside moiety was also showed the profound inhibitory effect on ASFV protease. These two flavonols apparently provide a way to develop anti-ASFV agents based on their scaffold.

## Introduction

African swine fever (ASF) is the most dangerous swine disease because of its large sanitary and socioeconomic impact. ASF generally has symptoms of high fever, inactivity, bleeding of the skin and internal organs and can result in death within 2–10 days, with mortality rates rising to 100%^1^. The ASF began in 1921, when it was first recorded in the Montgomery region of Kenya, Africa. Then, ASF has spread across the continent to not only Europe but also to Russia and more recently to Asia. Nowadays, ASF is pandemic in China and Hong-Kong^2–5^. It is threating to South Korea, too.

ASF is caused by the ASF virus (ASFV), a large, enveloped virus containing a 170–193 kbp double-stranded DNA encoding more than 150 genes. It is the only member of *Asfarviridae* family^6^. ASFV employs a polyprotein processing at Gly-Gly-Xaa sites to produce several core components of viral particles. The virus gene S273R encodes 31 kDa protein containing a “core areas” with the characteristics of SUMO-1 specific protease and adenovirus protease. The S273R product (ASFV protease) is known as the protease involved in the processing of the ASFV polyproteins pp220 and pp62. Therefore, ASFV protease is a good drug target for anti-ASFV infection^7^.

Recently, P1192R of ASFV has been proven to code for a functional type II DNA topoisomerase (ASFV-Topo II)^8^. The study of ASFV-Topo II with enrofloxacin suggested its key role both at intermediate and late stages of viral infection^9^. Since ASFV-Topo II plays an integral role in virus genome cloning and in the transcription process, this enzyme has been a target for the virus control. Topoisomerase poisons and inhibitors such as coumermycin A1, doxorubicin and amsacrine displayed a higher sensitivity against ASFV-Topo II^10^. A potent anti-ASFV effect of six fluoroquinolones also has been reported^11^.

In this study, we employed a proteolytic method to probe inhibitory compounds against ASFV protease. A synthetic peptide labelled with an EDANS-DABCYL FRET (Fluorescence resonance energy transfer) pair was used to search ASFV protease inhibitory compounds against a flavonoid library. Since the FRET pair was connected by a peptide including the ASFV protease recognition site, an increased intensity of fluorescence could be a sign to judge the presence of the catalytic activity of ASFV protease in this design.

With the FRET pair, a flavonoid library was screened to search ASFV protease inhibitory compounds. Recent studies showed that flavonoids have antiviral activity in some viruses including ASFV^12–14^. However, none of the antiviral studies with specific target protease at the molecular level has been reported. In this study, ASFV protease was selected as an antiviral target protein and assayed with a flavonoid library to find a potent inhibitory compound. The effects of flavonoids according to their scaffolds were analysed and the most promising flavonoid was suggested to be developed as a potent antiviral agent.

## Materials and methods

### Protein expression and purification

The coding sequence of pS273R, african swine fever virus (NCBI Ref. seq. NP_042804.1) was synthesised chemically by Bioneer (Daejeon, Korea) and cloned into a bacteriophage T7-based expression vector. The plasmid DNA was transformed into *E. coli* BL21 (DE3) for protein expression. *E. coli* BL21 (DE3) cells were grown on Luria–Bertani (LB) agar plates containing 150 *μ*g mL^−1^ ampicillin. Several colonies were picked and grown in capped test tubes with 10 mL LB broth containing 150 *μ*g mL^−1^ ampicillin. A cell stock composed of 0.85 mL culture and 0.15 mL glycerol was prepared and frozen at 193 K for use in a large culture. The frozen cell stock was grown in 5 mL LB medium and diluted into 1000 mL fresh LB medium. The culture was incubated at 310 K with shaking until an OD_600_ of 0.6–0.8 was reached. At this point, the expression of ASFV protease was induced using isopropyl-β-d-1-thiogalactopyranoside (IPTG) at a final concentration of 1 mM. The culture was further grown at 310 K for 3 h in a shaking incubator. Cells were harvested by centrifugation at 7650 *g* (6500 rev min^−1^) for 10 min in a high-speed refrigerated centrifuge at 277 K. The cultured cell paste was resuspended in 25 mL of a buffer consisting of 50 mM Tris–HCl pH 8.0, 100 mM NaCl, 10 mM imidazole, 1 mM phenylmethylsulfonyl fluoride (PMSF), 10 *μ*g mL^−1^ DNase I. The cell suspension was disrupted using an ultrasonic cell disruptor (Digital Sonifier 450, Branson, USA). Cell debris was pelleted by centrifugation at 24 900 g (15 000 rev min-1) for 30 min in a high-speed refrigerated ultra-centrifuge at 277 K.

The protein was purified by affinity chromatography using a 5 mL Hi-Trap His column (GE Healthcare, Piscataway, New Jersey, USA). The column was equilibrated with a buffer consisting of 50 mM Tris–HCl pH 8.0, 300 mM NaCl and 10 mM imidazole. The target protein was eluted with a buffer consisting of 50 mM Tris–HCl pH 8.0 and 100 mM NaCl with a gradient from 10 to 500 mM imidazole. The purified protein was buffer exchanged into 20 mM Tris pH 7.5 using Vivaspin 20 MWCO 10 kDa (GE Healthcare), a centrifugal device. SDS–PAGE showed one band around 32 kDa (31550.22 Da), corresponding to the molecular weight of ASFV protease. The protein was concentrated to 1.38 mg mL^−1^ for the protease assay in a buffer consisting of 20 mM Tris pH 7.5.

### FRET protease assays with ASFV protease

The custom-synthesised fluorogenic substrate, DABCYL-KVGGNETQ-EDANS (ANYGEN, Gwangju, Korea), was used as a substrate for the proteolytic assay using ASFV protease^7^. This substrate contains Gly-Gly-Xaa site and the proteolytic cleavages occur after the second Gly of the consensus sequence Gly-Gly-Xaa which is recognised as a cleavage site in the maturation of the protein. The peptide was dissolved in distilled water and incubated with each protease. A SpectraMax i3x Multi-mode microplate reader (Molecular Devices) was used to measure spectral-based fluorescence. The proteolytic activity was determined at 310 K by following the increase in fluorescence (λ_excitation_ = 340 nm, λ_emission_ = 490 nm, bandwidths = 9, 15 nm, respectively) of EDANS upon peptide hydrolysis as a function of time. Assays were conducted in black, 96-well plates (Nunc) in 350 *μ*L assay buffers containing protease and substrate as follow; For the ASFV protease assay, 8 *μ*L of 0.044 mM protease containing 20 mM Tris pH 7.5 was incubated with 3.5 *μ*L of 0.1 mM substrate at 310 K for 2 h before measuring Relative Fluorescence Units (RFU). Before the assay, the emission spectra of 65 flavonoids were surveyed after illuminating at 340 nm to avoid the overlapping with the emission spectrum of EDANS. Every compound was suitable to be tested. The final concentration of the protease, peptide and chemical used at the assay was 1 *μ*M, 1 *μ*M and 20 *μ*M each. At first, ASFV protease and chemical were mixed and preincubated at room temperature for 1 h. The reaction was initiated by the addition of the substrate and each well was incubated at 310 K for 2 h. After that, we measured the fluorescence of the mixture on the black 96-well plate using the endpoint mode of SpectraMax i3x where the excitation wavelength was fixed to 340 nm and the emission wavelength was set to 490 nm using 9, 15 nm bandwidth, respectively. All reactions were carried out in triplicate. Among the first sixty-five flavonoids (Supplementary Table 1), one of them was picked up to further assay at a concentration range of 1 *μ*M ∼ 40 *μ*M. The IC_50_ value which is the value causing 50% inhibition of ASFV protease was calculated by nonlinear regression analysis using GraphPad Prism 7.03 (GraphPad Software, San Diego, CA, USA).

### FRET protease assays with ASFV protease in the presence of triton X-100

The proteolytic assay using ASFV protease in the presence of Triton X-100 has been performed to differentiate the artificial inhibitory activity of chemicals through non-specific binding with proteases by forming aggregate or complexation. The concentration used in this study was 0.01%.

### Absorption spectroscopic studies based on tryptophans of ASFV protease

To confirm the feasibility of the assay method independently, the fluorescence spectra from the tryptophan of ASFV protease with candidate inhibitors were investigated^15^. The fluorescence measurements were recorded with a SpectraMax i3x Multi-mode microplate reader (Molecular Devices) at excitation and emission wavelengths of 290 nm and 300–500 nm, respectively. The optimal excitation and emission wavelengths were determined by SoftMax Pro. Four tryptophan of ASFV protease showed a fluorescence emission with a peak at 350 nm after the excitation at the wavelength of 290 nm. In contrast, the flavonoids were almost non-fluorescent under the same experiment condition. Each 20 *μ*M and 40 *μ*M chemical was incubated with 1 *μ*M ASFV protease for 1 h and the fluorescence intensity of the mixture was measured.

## Results

The cell yield harvested for purification of ASFV protease was 3.1 g per 2000 mL of *E. coli* culture. The amount of purified protein synthesised with His_6_-tag was 2.76 mg. For storage and assay, the protein solution was concentrated to 1.38 mg mL^−1^. The concentrate was diluted to 1 *μ*M when the inhibitory assay was carried out.

**Figure 1.**
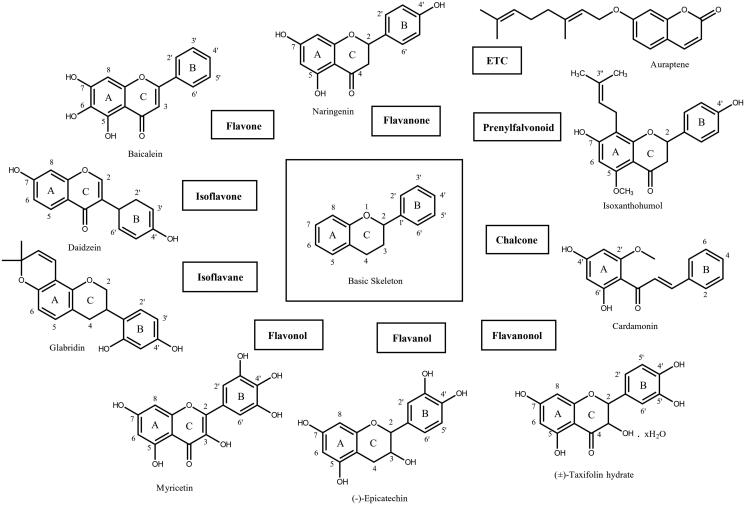
The example skeleton structures of flavonoids. Ten different scaffolds classified in this study were displayed with example flavonoid derivatives. Basic skeletons and their carbon atoms numbering pattern were drawn.

**Figure 2.**
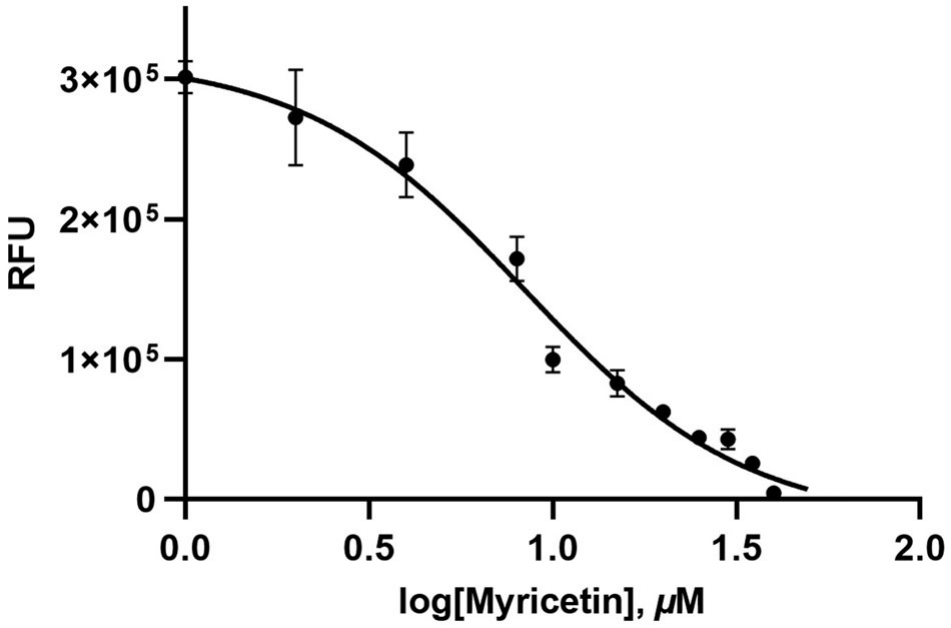
Results from the FRET method. Each data point represents the effect of myricetin against ASFV protease compared to the control. The RFU are plotted against the log-concentration of inhibitory compounds. Each dot is expressed as the mean ± standard error of the mean (*n* = 3). RFU: Relative Fluorescence Units.

A flavonoid library consisting of ten different scaffolds was also built (Figure 1). It contains five isoflavones, one isoflavane, seventeen flavones, twelve flavonols, seven flavanols, seven flavanones, four flavanonol, one prenylflavonoid, nine chalcones and two unclassified flavonoids (Supplementary Table 1). We applied the library to assay ASFV protease. Using sixty-five flavonoids, an inhibitory effect of each compound at 20 *μ*M was tested. Among them, myricetin (3,3′,4′,5,5′,7-Hexahydroxyflavone) were found to have prominent inhibitory activity. The binding affinity data were plotted as log inhibitor concentration versus percent fluorescence inhibition (Figure 2). The compound showed the severely reduced fluorescent intensity and thus represented their ASFV protease inhibitory activity. The IC_50_ value was calculated from the dose-dependent inhibitory curve of myricetin. The measured values were 8.4 *μ*M. Since flavonoids are known to be aggregated through complexity and thus non-specifically inhibit various proteases, the assay in the presence of Triton X-100 was also performed^16^. Before the examination, we tested the effects of Triton X-100 on ASFV protease. As shown in Supplementary Figure 1, only a slight increase in catalyst activity was observed up to 0.1% Triton X-100. Therefore, the assay was performed at a concentration of 0.01% Triton X-100 with no significant interference detected.

**Figure 3.**
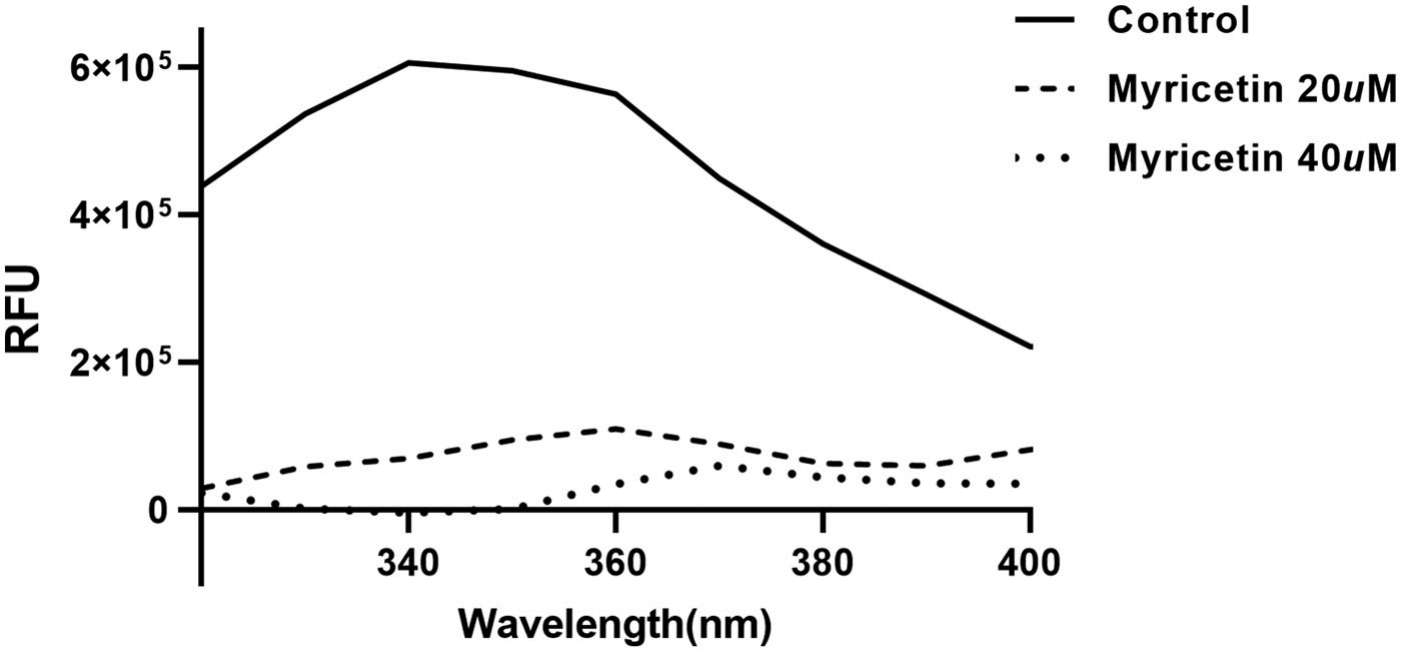
Fluorescence quenching spectra of ASFV protease. A solution containing 1 *μ*M ASFV protease showed a strong fluorescence emission (the solid line) with a peak at 340 nm at the excitation wavelength of 295 nm. After adding 20 *μ*M (the dashed line) and 40 (one dotted line) *μ*M inhibitory compound, fluorescence quenching spectra were obtained.

To independently confirm the inhibitory activity of flavonoids, a general tryptophan based assay method was employed. Tryptophan was well known to emit its fluorescence. Therefore, if tryptophan is positioned adequately in proteins, the change of fluorescence intensity can reflect the binding state of chemicals and be used to judge the interaction between proteins and chemicals. The ASFV protease contains four tryptophan residues. Therefore, its fluorescence change can reflect the environmental variation of the protein. The ASFV protease used in this study displays a fluorescence peak at 340 nm after the tryptophan excitation wavelength of 295 nm. We monitored the change of the fluorescence intensities depending on the presence or absence of all flavonoids. Since each compound in the flavonoid library was almost non-fluorescent under the experiment condition, a change of fluorescence intensity reflects interactions between the protein and chemical. The decreased emission intensity confirmed the complex formation between the ASFV protease and the inhibitory compound (Figure 3).

## Discussion

ASFV has caused devastating problems from European continent in 2007 to Asian continent now^2–5^. Pig farmers desperately tried to prevent the spread of ASFV and the economy of affected countries was negatively influenced. Considering the absence of vaccine, the development of therapeutic agents is crucial to prevent pandemic of ASFV. Flavonoids belong to a class of plant secondary metabolites with a polyphenolic structure widely found in fruits and vegetables. They have a wide range of biochemical and pharmacological effects including antioxidants, anti-inflammatory, anti-mutagens and anti-cancer-causing properties combined with the ability to control major cellular enzyme functions. Intriguingly, some flavonoids also have antiviral activity^17^. Specifically, ASFV was reported to be inhibited by apigenin^12^ and genkwanin^14^ and thus its infection in Vero cells was severely reduced. The former affected on its protein synthesis and viral factory formation and the latter for its entry and egress stages. In other cases such as HCV NS3 serine protease and dengue‐2 virus NS3 protease, main proteases responsible for viral processes were inhibited directly by some flavonoids^18^^,^^19^. Therefore, ASFV protease can be a promising target to suppress the pathogenicity of ASFV.

**Figure 4.**
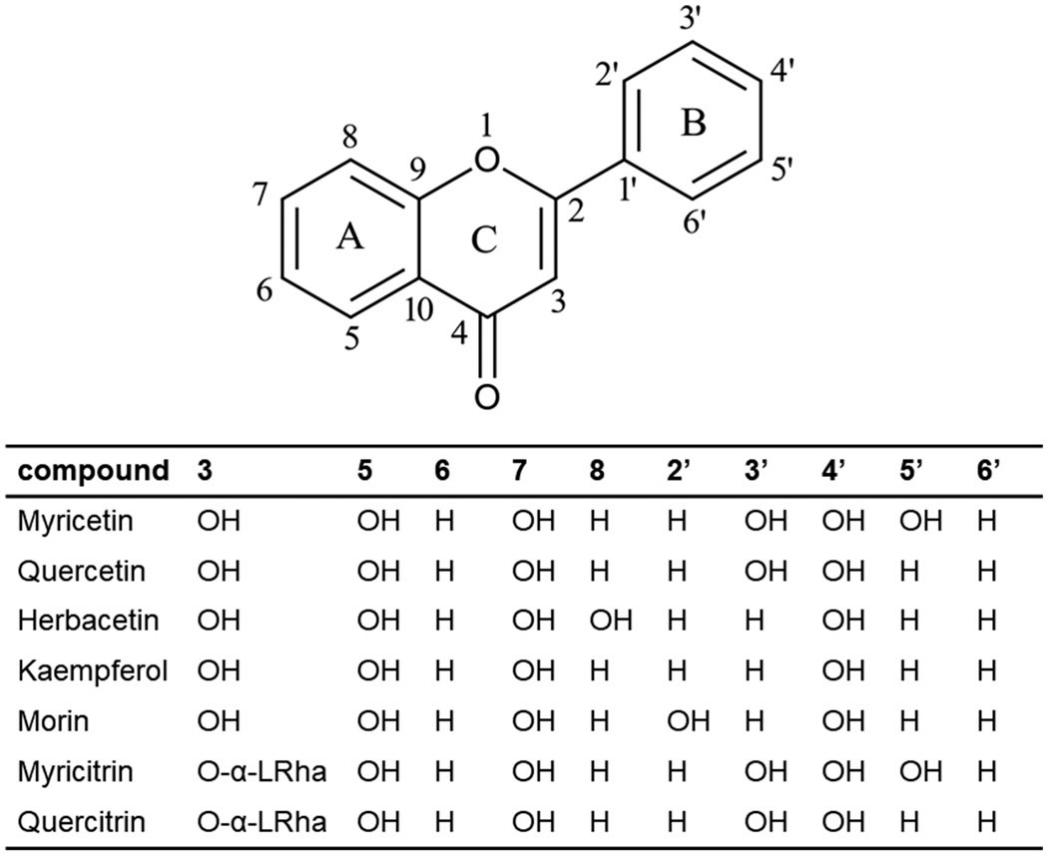
Schematic representation of flavonols. Structures of inhibitory compounds discussed in this study were listed and the positions of substituted hydroxyl groups were demonstrated. Since they belong to the flavonol family, the hydroxyl group at the carbon atom position 3 is conserved. In myricitrin and quercitrin, the L-rhamnoside moiety was placed instead of the hydroxyl group.

**Figure 5.**
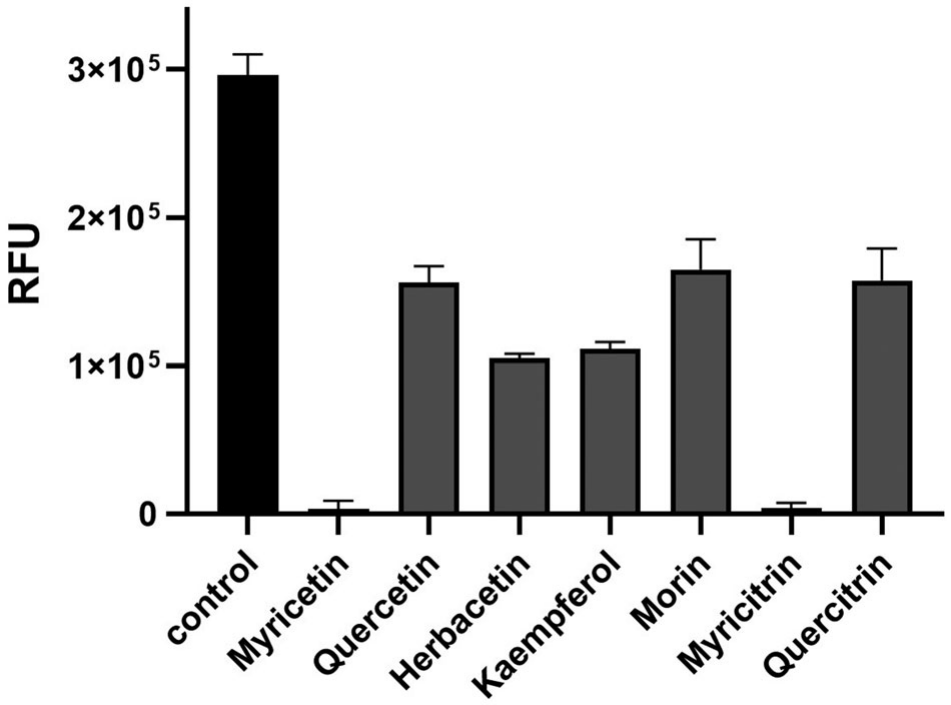
The effects of flavonoids on the ASFV protease activity. Each bar represents the inhibitory activity of compounds with 0.01% Triton X-100. The first black bar represents the control. Inhibitory compounds were used at 40 *μ*M concentration. Each bar is expressed as the mean ± standard error of the mean (*n* = 3). RFU: Relative Fluorescence Units.

In this study, the systematic analysis using various scaffolds of flavonoids targeting ASFV protease was performed. In order to find the best scaffold to inhibit the function of ASFV protease, an assay with various flavonoid derivatives classified in ten scaffolds was built and performed. Among them, the flavone, chalcone and flavonol scaffolds displayed potential inhibitory effects in order. Although the backbone skeleton of flavonol is different from that of chalcone, it is quite similar to that of flavone except the presence of the additional 3-hydroxyl group in its chromen-4-one ring (Figure 1). Since the inhibitory activity is clearly better with flavonol than flavone derivatives, the 3-hydroxyl group of flavonol seems to contribute to interact with ASFV protease (Figure 4). The best inhibitory compound was myricetin with the flavonol scaffold (Figure 2). The comparison with other homologues showed that the three hydroxyl groups of the 3,4,5-trihydroxylphenyl ring of myricetin is essential for its inhibitory activity. For example, quercetin with a 3,4,-dihydroxylphenyl ring clearly showed diminished activity. Its activity, however, is similar to those of herbacetin, kaempferol and morin (Figure 5). It implies that the 3,4,5-trihydroxyphenyl group of myricetin is important for its inhibitory function.

In order to find out better inhibitory derivatives of myricetin, a commercially available compound, myricitrin, was additionally investigated. IC_50_s of myricetin and myricitrin were 8.4 *μ*M and 2.7 *μ*M, respectively. The extra rhamnoside of myricitrin might perform a positive contribution for interaction to ASFV protease. The closest myricitrin homologue, quercitirn, which also contains the extra rhamnoside was reviewed. Interestingly, quercitirn did not showed prominent inhibitory activity. The comparison again confirmed the importance of 3,4,5-trihydroxyphenyl group of myricetin by representing its better affinity (Figure 5).

The natural polyphenol found with hydroxyl groups at 3, 5, 7, 3′, 4′ and 5′ positions of flavonol, myricetin, is very common in berries, vegetables, and in teas and wines produced from various plants. It may possess anticancer activity against hepatic, skin, pancreatic and colon cancer cells. It also has beneficial biological functions such as anti-inflammatory, anti-hypertensive properties^20^, and anti-HIV activities^21^. We first discovered and reported its potential anti-ASFV activity. Myricetin and its derivative, myricitrin, clearly suggested that their basic skeleton can be used as a reference scaffold to develop better chemicals as anti-ASFV agents. Unfortunately, a crystal structure of ASFV protease is not yet been known. Therefore, a further study is going on to determine the X-ray crystal structure of ASFV protease together with its complex structures with myricetin derivatives.

## Conclusion

We formed a flavonoid library to systematically investigate ASFV protease inhibitory compounds by FRET method. Among them, the flavone, chalcone and flavonol scaffolds produced potential inhibitory effects in sequence. Myricetin and its derivative, myricitrin, with the flavonol scaffold were the best inhibitory compounds against ASFV protease in the flavonoid library. The binding of the flavonoids was independently demonstrated by a tryptophan-based fluorescence method. The three hydroxyl groups of the 3,4,5-trihydroxylphenyl ring of myricetin are essential for its inhibitory activitie and the additional rhamnoside of myricitrin can positively contribute to the interaction with ASFV protease. Since the crystal structure of ASFV protease is not yet known, further research will be conducted to determine the X-ray crystal structure of ASFV protease with myricetin derivatives.

## Supplementary Material

Supplemental Material
