# Supplementary material for: Inhibition of African swine fever virus protease by myricetin and myricitrin
Source: J Enzyme Inhib Med Chem. 2020 Apr 17;35(1):1045–9. doi: 10.1080/14756366.2020.1754813 (PMC7178854; doi:10.1080/14756366.2020.1754813)
Supplement: Supplemental Material [file IENZ_A_1754813_SM1386.zip › IENZ_A_1754813_SuppTable.pdf]

Supplementary Table 1. A flavonoid library

No	Name of compound	No	Name of compound
1	Daidzein	34	Icaritin
2	Genistein	35	(−)-Gallocatechin
3	Genistin	36	(±)-Epigallocatechin gallate
4	Ipriflavone	37	(−)-Epicatechin
5	Puerarin	38	(±)-Catechin
6	Glabridin	39	(−)-Gallocatechin gallate
7	Baicalein	40	(−) Catechin gallate
8	Diosmin	41	(+)-Catechin hydrate
9	Diosmetin	42	Hesperidin
10	Skullcapflavone II	43	Naringenin
11	beta-Naphthoflavone	44	Sakuranetin
12	Orientin	45	Naringin
13	Acacetin	46	Poncirin
14	Baicalin	47	Bavachin
15	Rhoifolin	48	Flavanone
16	Hispidulin	49	(±)-Taxifolin hydrate
17	Sinensetin	50	Silibinin
18	Oroxin B	51	Astilbin
19	Pectolinarin	52	Silymarin
20	Cirsiliol	53	Isoxanthohumol
21	Homoplantaginin	54	Isobavachalcone
22	Amentoflavone	55	2,2′,4′-Trihydroxychalcone
23	Luteolin	56	Dienestrol
24	Herbacetin	57	Sofalcone
25	Kaempferol	58	Rhodamine 6G
26	Morin	59	FCLA Free Acid
27	Myricetin	60	Helichrysetin
28	Fisetin	61	Cardamonin
29	Quercitrin	62	Neohesperidin dihydrochalcone
30	Quercetin	63	Mangiferin
31	Quercetin 3-β-D-glucoside	64	Auraptene
32	Kaempferol 7-O-β-D-glucopyranoside	65	Myricitrin
33	Rutin		

[*]Isoflavone;1-5, Isoflavane;6, Flavone;7-23, Flavonol;24-34 & 65, Flavanol;35-41, Flavanone;42-48,

Flavanonl;49-52, Prenylflavonoid;53, Chalcone;54-62, unclassified;63-64
