# Supplementary figures and images for: Inhibition of African swine fever virus protease by myricetin and myricitrin
Source: J Enzyme Inhib Med Chem. 2020 Apr 17;35(1):1045–9. doi: 10.1080/14756366.2020.1754813 (PMC7178854; doi:10.1080/14756366.2020.1754813)

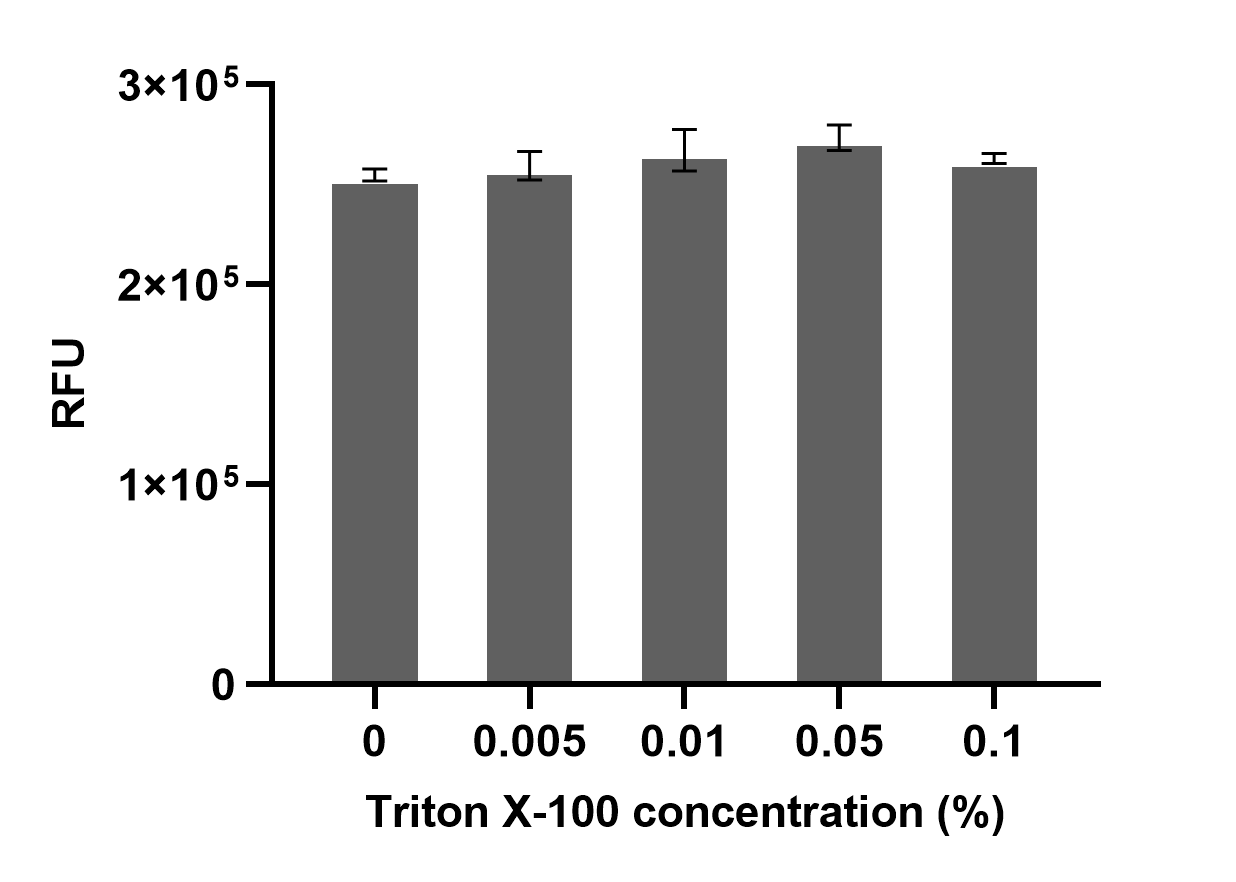

Supplement: Supplemental Material [file IENZ_A_1754813_SM1386.zip › IENZ_A_1754813_SuppFigure1.tif]
